# Molecular Rotors as Switches

**DOI:** 10.3390/s120911612

**Published:** 2012-08-27

**Authors:** Mei Xue, Kang L. Wang

**Affiliations:** Device Research Laboratory, Department of Electrical Engineering, University of California, Los Angeles, CA 90095, USA; E-Mails: mxue@ee.ucla.edu (M.X.); wang@ee.ucla.edu (K.L.W.)

**Keywords:** molecular rotor, molecular devices, switching, memory, crossbar architecture

## Abstract

The use of a functional molecular unit acting as a state variable provides an attractive alternative for the next generations of nanoscale electronics. It may help overcome the limits of conventional MOSFETd due to their potential scalability, low-cost, low variability, and highly integratable characteristics as well as the capability to exploit bottom-up self-assembly processes. This bottom-up construction and the operation of nanoscale machines/devices, in which the molecular motion can be controlled to perform functions, have been studied for their functionalities. Being triggered by external stimuli such as light, electricity or chemical reagents, these devices have shown various functions including those of diodes, rectifiers, memories, resonant tunnel junctions and single settable molecular switches that can be electronically configured for logic gates. Molecule-specific electronic switching has also been reported for several of these device structures, including nanopores containing oligo(phenylene ethynylene) monolayers, and planar junctions incorporating rotaxane and catenane monolayers for the construction and operation of complex molecular machines. A specific electrically driven surface mounted molecular rotor is described in detail in this review. The rotor is comprised of a monolayer of redox-active ligated copper compounds sandwiched between a gold electrode and a highly-doped P^+^ Si. This electrically driven sandwich-type monolayer molecular rotor device showed an on/off ratio of approximately 10^4^, a read window of about 2.5 V, and a retention time of greater than 10^4^ s. The rotation speed of this type of molecular rotor has been reported to be in the picosecond timescale, which provides a potential of high switching speed applications. Current-voltage spectroscopy (I-V) revealed a temperature-dependent negative differential resistance (NDR) associated with the device. The analysis of the device *I–V* characteristics suggests the source of the observed switching effects to be the result of the redox-induced ligand rotation around the copper metal center and this attribution of switching is consistent with the observed temperature dependence of the switching behavior as well as the proposed energy diagram of the device. The observed resistance switching shows the potential for future non-volatile memories and logic devices applications. This review will discuss the progress and provide a perspective of molecular motion for nanoelectronics and other applications.

## 1. Introduction

Miniaturization has been one of the driving forces of the semiconductor industry based on the well-known Moore's law since Richard Feynman stated in his famous talk ‘there is plenty of room at the bottom’ in 1959. However, as the physical limitation comes closer, further scaling of the basic components of the microelectronic circuit such as memory and switch are now being questioned. The anticipation of this limitation has led researchers in academia and industry alike to consider many conceptually new materials and structures from the molecular scale point of view. The above scenario provides an opportunity for exploring molecular machines and nanomachines which can be functionalized as nanoscale electronic devices. These devices based on functional molecular units acting as state variables (e.g., rotation and motor) are attractive due to their potential ultimate small size, low-cost, low variability, highly integratable characteristics and the ability to self-assemble themselves [1–6].

A specific kind of molecular machine can be a particular molecular device designed to perform mechanical movements under control of appropriate energy inputs [7,8]. Triggered by external stimuli such as light, electricity or chemical reagents, these molecular devices have shown various functions including diodes [9], rectifiers [1,3], memories [10–12], resonant tunnel junctions [13–24] and single settable molecular switches that can be electronically configured for logic gates [25–37]. In general, however, a molecular machine should be capable of performing an action leading to a desired function following the input of a signal with a supply of energy. Recent demonstrations of wholly synthetic molecular machines include rotary motors [38,39], a rotary motor displaying net unidirectional motion [40], and linear motors [41,42]. There are reports on the use of rotaxane for crossbar memory in which the linear motion is introduced by redox in solution and in solid support [42]. There are many aspects of different emphases of recent molecular rotor development, including design of structures [43–48], stimulus [49–51], speed [52], and transport [53], *etc.* The focus of discussion here is on potential devices which could be integrated with conventional semiconductor technologies.

In this paper, we limit our discussion to the rotational motion and we first describe the different types of rotary motors generally. Then we will focus on a specific electrically driven surface mounted molecular rotor because of its potential capability of integration with standard semiconductor fabrication technologies, which provides the realization possibility in the near future. In particular, we will discuss their molecular structures, mechanisms and operation principles, respectively. Finally we will provide a perspective of scalability, challenges and potential applications. This review will concentrate on a real prospect of integrating a bottom-up approach, based on molecular design and micro-fabrication, in order to construct molecular electronic devices that can store information with very high densities.

### 1.1. Molecular Rotor as Molecular Switches

Besides the linear motion, as mentioned in the previous section, the rotary motion is another category of molecular machines. Like the linear motion of molecules, these molecules can be activated to make rotation movement chemically, optically, or electrically as a switch, and the recognition of the change of positions of the chemical elements by the charge transport through the layer results in the off and on states.

The molecular rotor usually consists of two parts that can easily rotate relative to each other. The different types of rotors can generate different rotation angles due to the specific rotating ligands. Normally the part with a larger moment of inertia is defined as the stator and the part with a smaller moment of inertia as the rotator, while the term rotor refers to the whole molecule. The distinction is somewhat arbitrary except the stationary part is fixed on or sits within a much more massive object, or on a solid substrate. In the absence of a fixed mounting, the rotator and the stator both turn around the same axle.

Showing proper electrical characteristics, some molecule rotors foray into the new field of molecular electronics. The idea is the use of molecular building blocks for the construction of electronic components, both passive (e.g., resistive wires) and active (e.g., transistors). The concept of molecular electronics is attractive and excites scientists and engineers alike due to its prospect of size reduction in electronics offered by molecular-level control of assembly. Hence it provides means to go beyond the anticipated physical size of the conventional CMOS in view of fundamental limiting processes (e.g., tunneling) and cost of top down micro-fabrication technology. However, to realize logic functions using molecular devices, we may face the challenges that the dynamics of molecules in contact with electrodes is far from easy grasp in determining its mechanism as the effects of electrodes and interfaces and others tend to complicate the characterization of the behaviors. In particular, the effect of different switchable and nonswitchable compounds on the observable electrical signals, such as current flow and voltage thresholds, complicate the problem and thus has to be carefully studied to obtain a microscopic understanding of what is really happening inside a particular molecular electronic device. Consequently, appropriate control experiments need to be performed and great care must be exercised in order both to understand the principle of operation and to attribute specific models to the observed switching effects. Although in many cases, results obtained and conclusions drawn may not be as definitive as those of traditional semiconductor physics, where analytical techniques have been relatively mature, hypothesis testing and detailed investigation can help further fundamental understandings.

Molecule-specific electronic switching has been reported for several device structures, including nanopores containing oligo(phenylene ethynylene) monolayers [11,12] and planar junctions incorporating rotaxane and catenane monolayers [9,10]. One of the most common and simplest structures of these molecular electronic devices is a crossbar of two electrodes (either two metal electrodes or a metal electrode and a highly doped Si substrate), between which a molecular switch is sandwiched. One example is that an electrically switchable molecule—a bistable catenane—was placed between a Si bottom electrode, covered by its native oxide, and a Ti/Au top electrode [43]. Although these molecular devices offer excellent scaling potential, high ON/OFF ratio and adequate programming speed still need to be demonstrated. Alternative to molecular structures, some efforts have also been focused toward the fabrication of nanowires with a wide variety of structures including doping levels, lengths, widths, concentric tings, and alternating materials because of their elegant electronic functions [54,55], these nanowire-based devices, however, are lack of the rich mechanical structure inherent in a molecule.

Molecular switches use a state variable for which information storage relies on physical changes of the molecule. For example, a bistable molecular switch may be immobilized onto a bottom electrode and the molecular layer is then sandwiched by a top metal electrode (*i.e.*, macroscopic gold electrodes) to achieve its different states. In particular, transition metals offer useful features in controlling these state variables due to their accessible oxidation states. This process can results in low power consumption. The variables which can be controlled in the transition metals include coordination number, color, and geometry [56]. Metal complexes of copper [57–62], nickel [63,64], and platinum can have different geometries in different oxidation states, and the different conformations from the different oxidant states can be used as a state variable, *i.e.*, the metal in the center can be used to drive intra-rotational motion. The next section will address rotational molecular switches.

### 1.2. Classification of Molecular Rotors

**Figure 1.**
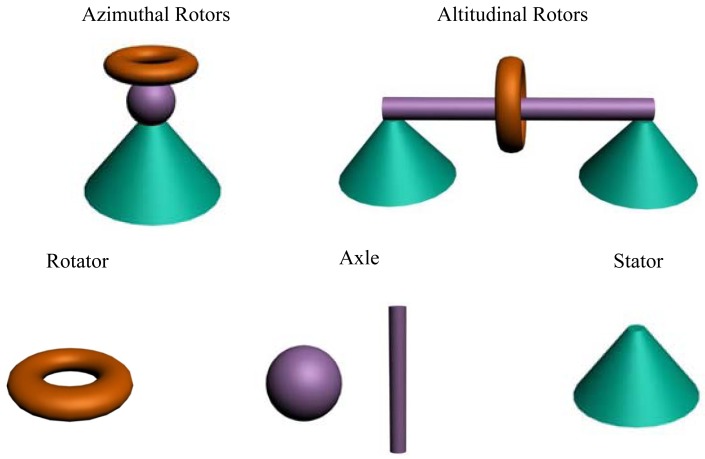
Classification for surface mounted molecular rotors showing azimuthal and altitudinal rotors. Each component make up the rotor is shown below.

There is no common rule to classify molecular rotors. Various criteria have been used for the classification, such as phase base, rotation angle, and molecular compounds. In this review, we use the phase based method. We divide rotors into three categories: solution phase, solid phase, and surface mounted molecular rotors. A solution phase molecular rotor is the one that can float freely in solution or vapor; a solid phase molecular rotor is the one that is located (but not attached to the substrate) on a solid support; and the surface mounted molecular rotor is that is attached or bonded to the surface of a solid support. As discussed above, for solution phase rotors, the distinction between the rotator and the stator may be sometimes ambiguous, as both parts of the molecule rotator are related to each other. For solid phase rotors, the molecule close to the solid support is normally the stator. And, for a surface-mounted molecule, one can clear specify the stator, which is rigidly attached to the surface, and forms a part of the molecular structure. In addition, the axle of rotation about which the rotator turns can be perpendicular (an azimuthal rotor) or parallel (an altitudinal rotor) to the surface (Figure 1). For solid form of molecular rotors, the molecule component usually consists of a rotary part and a structure which separates adjacent rotators.

Within each category of solution phase, solid phase and surface mounted molecular rotors; a subdivision may be made based on the chemical nature of the axle, such as a single bond, a triple bond, or a metallic atom. This chemical nature of the bond strongly influences one of the fundamental energy terms in the system, the intrinsic barrier energy and width of rotation.

In addition to the above structural classification, the other method to categorize rotors is by their nature of dynamics under a given set of environmental conditions. Two extreme cases of thermally activated systems are: (i) the rotor with a small rotational potential energy barrier such that it is easy for the rotor to reorient at a sufficient temperature; and (ii) those having a stator lying in the lowest energy state and thus a large barrier. Switching will normally not occur unless the system is perturbed by an external force. It is obvious that the interplay of the different energy terms in a system determines the rotor dynamics; the environment temperature also plays an important role in determining the energy barrier of rotation.

## 2. Solution Phase Molecular Rotors

Previously reported rotary motions such as a rotational actuator based on multi-walled carbon nanotubes [65], fluorescent molecular rotors [66], and most types of rotary device [67], operate most often in solution environments.

**Figure 2.**
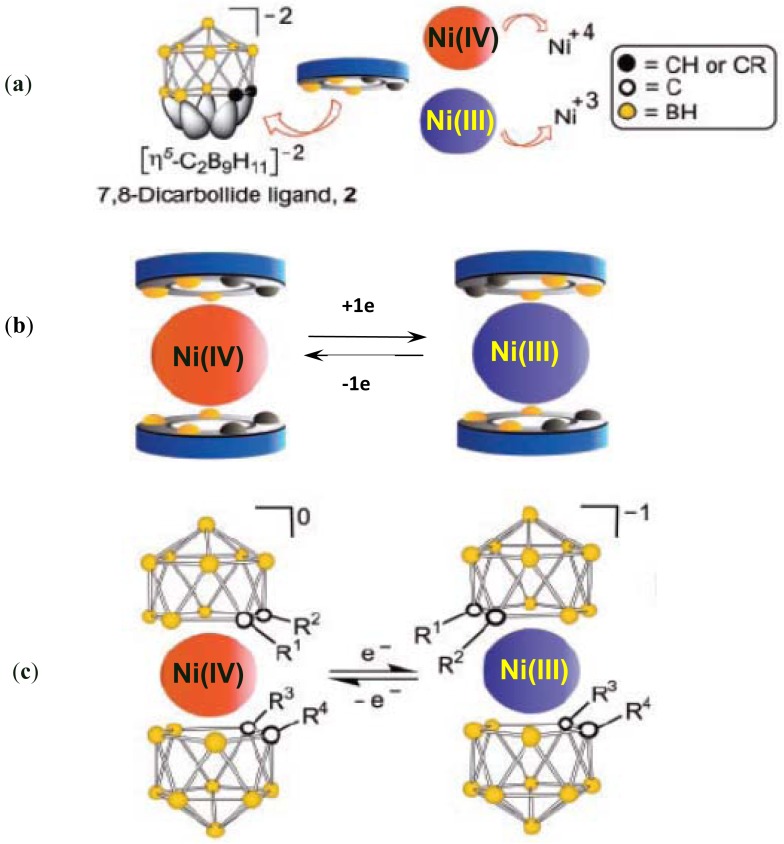
Illustration of rotor configurations of Ni(IV) and Ni(III) and the structural modules from which they are constructed. Structure Ni(III) is defined with rotation angle α = 0°. (**a**) Representative of each symbol in the schematic figure; (**b**) Schematic of the transition between Ni(IV) and Ni(III); (**c**) Detail structure of the transition between Ni(IV) and Ni(III). The diastereomers obtained by substitution of R for H at a single carbon vortex of the 7,8 ligands are meso (R^1^=R^4^=H, R^2^=R^3^=substituent) and dl (R^1^=R^3^=H, R^2^=R^4^=substituent and R^1^=R^3^=substituent, R^2^=R^4^=H; d and l assignments are arbitrary) [68].

A typical example is a molecular rotor comprising of a carborane cage ligand (7,8-dicarbollide) around a nickel axle [68]. The Ni(III) metallacarborane structure is a *transoid* sandwich with two pairs of carbon vertices reflected through a center of symmetry, but that of the Ni(IV) species is *cisoid* (see Figure 2). The interconversion of the two provides the basis for controlled rotational and oscillatory motion.

The schematic structure of this molecular device is shown in Figure 2. And the fundamental mechanism has been known since the discovery [69] of the d7 Ni(III) and d6 Ni(IV) *commo*-bis-7,8-dicarbollyl metallacarboranes, denoted as Ni(III) [70] and Ni(IV) [71] (Figure 2); These complexes are produced by the coordination of two dicarbollide ions [72] with a Ni(II) ion, followed by subsequent oxidation. The rotation may happen between ligands of this species with a three times higher rotational barrier energy than that of the well-known metallocenes [73,74].

The interconversion of the geometries of Ni(III) and Ni(IV) controlled by the change of the nickel oxidation state provides the basis for the controlled, rotational, oscillatory motion, and can be achieved electrochemically by redox reactions or photochemically. It is based on the presence of two adjacent CH vertices, which introduces localized regions of reduction negative charge [75]. The resulting inter-ligand interaction leads to two configurations. Most of examples is a *trans* configuration [70,72,76] (such as in Ni(III)) in *commo*-bis-7,8-dicarbollyl metallacarboranes, while some forms the *cisoid* configuration (such as in Ni(IV)) with its pairs of carbon vertices on the same side of the molecule [69,70,77]. Thus, an oscillatory molecular rotor, providing useful work on the molecular scale, is possible based on the controlled 4π/5 rotation of the dicarbollide ligands coupled to an ancillary structure. Based on the previous studies of the electronic and infrared spectra, the authors believe that both Ni(III) and Ni(IV) maintain their solid-state structures [70,71] in solution. Thus they demonstrated the quantitative reversibility of the redox couple using reversible cyclic voltammograms (–0.66 V *versus* the standard calomel electrode (SCE)). The authors also showed experimentally and theoretically that the interconversion acts as a rotating molecular machine in their paper. In their model, the photoexcitation induced geometrical changes were simulated to compare with the results caused by electrochemical reduction. Therefore reduction places an electron in the lowest unoccupied molecular orbital (LUMO); photoexcitation removes an electron from the highest occupied molecular orbital (HOMO) and places it in the LUMO. There are other solution phase molecular rotors. For example, in suitably designed rotaxanes, the swinging movement of the ring around the axle can be electrochemically driven as well.

## 3. Solid Phase Molecular Rotors

**Figure 3.**
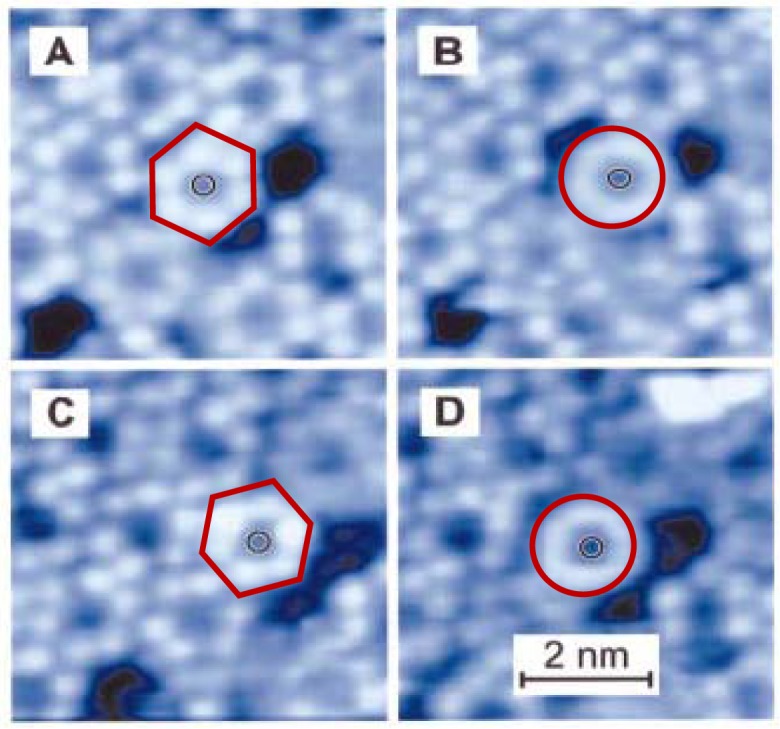
Sequence of STM images of an atomically clean Cu(100) surface after exposure to a coverage just below one complete monolayer of HB-DC measured in UHV at room temperature. In (**A**) and (**C**), the same molecule is translated by 0.26 nm and imaged as a six-lobed structure in registry with the surrounding molecular layer. Image area is 5.75 nm by 5.75 nm, recorded with a tunnel voltage of *V*_t_ = 0.35 V and a tunnel current of *i*_t_ = 90 pA [78]. In (**B**) and (**D**) the molecule is imaged as a torus and is in a location where it is not in phase with the overall 2D molecular overlayer (disengaged state).

As we mentioned above, the solid phase molecular rotor is located (but not attached to the substrate) on a solid support. A good example is that of single-molecule rotors, and it was found that the rotor, from a study of scanning tunneling microscopy (STM) in ultrahigh vacuum [78], was surrounded by a set of molecules that forms a supremolecular bearing on a surface as illustrated in Figure 3. Such molecular rotors are propeller-shaped hexa-*tert*-butyldecacyclene (HBD) molecules of around 1.5 nm in diameter (Figure 3). It shows that the HBD was deposited on an atomically clean Cu(100) surface. For a surface coverage of slightly less than one monolayer, a close-packed supramolecular layer with nanometer sized holes is formed by the molecules. As a result of robust intermolecular interactions the packed HBD molecules cannot rotate on the plane of the Cu(100) surface and they appear in the STM images as six-lobed objects as in Figure 3. Some of these molecules can, however, dissociate from the supramolecular assembly to enter one of the nanometer sized voids (Figure 3 dark areas) in which they are free to rotate. The rate of rotation is greater than the scan rate of imaging at ambient temperature and, as a result, the molecules in motion look like toroidal objects (Figure 3(B,D)). Interestingly, a single rotating molecule can be translated, with the aid of the STM tip, to a position where it is immobilized by the surrounding molecular layer (Figure 3(A,C)). It should be noticed that, although this system represents an impressive example of observation of molecular motion, it cannot be considered as a molecular machine because the motor was the result of external manipulation. Alternative means to control the motion is needed.

Supported by experiments of this kind and, by the existence of a wealth of nanometer scale natural molecular machines [79,80], it seems reasonable to extend general macroscopic concepts of artificial machines to the molecular level [81,82]. In doing so, however, cautions must be exercised because molecular machines, particularly natural ones, like to operate at energies only marginally higher than that of the thermal bath and hence can be subjected to large fluctuations, and thus carefully engineered mechanisms may be devised with proper energy structure to assure the stability of the motions.

## 4. Surface Mounted Molecular Rotors

As we mentioned in the section of solution phase molecular rotors, previously reported rotary motions including a rotational actuator [65,83], fluorescent molecular rotors [66], rotation of a single molecule within a supramolecular bearing [84], operate most often in solution environments. However, in the solution state, rotational motions are often subjected to a statistically random distribution of active components, and thus are difficult to be expressed coherently in an electrical context. It becomes imperative to immobilize and organize these nanoscale rotors onto a solid support in order to limit degrees of freedom while bringing coherence and cooperativity to bear out their coordinated performance. Clearly, mechanical switching must be shown when the active molecular rotors are arranged in a closely packed monolayer on a solid support.

The use of transition metal ions as templates to construct multicomponent chemical systems with interlock or knotted topologies has been extensively exploited [85]. Some of these species constitute nice prototypes of nanomachines that can be operated by chemical, electrochemical reaction as well as photochemistry [86]. Silicon-immobilized heteroleptic copper compounds are attractive as nanoswitches because they exhibit a bistable rotational mechanical motion by switching the oxidation state of the copper metal center. This one electron redox-induced conformational change is reversible and occurs via the +1 and +2 oxidation states of copper, which isomerizes from the tetrahedral to square planar geometries, respectively. The ionization potential of the copper switch constitutes the minimum energy required for information storage *i.e.*, eV_s_ per molecule, where V_s_ is the voltage for which the redox event occurs. Because information storage is based on the nanoscopic movement of atomic nuclei about the copper center, the maximum rate of switching process is fundamentally limited by the frequencies of the relevant rotational vibrational modes of the compound, giving a minimum switching speed on the order of a picosecond [87]. The heteroleptic design is required for a bidentate ligand to immobilize onto a silicon substrate while the second ligand is used to perform useful work. With a silicon substrate, the molecular nanoswitches can be potentially integrated with the conventional CMOS circuit to achieve logical functions and system-on-chip.

**Figure 4.**
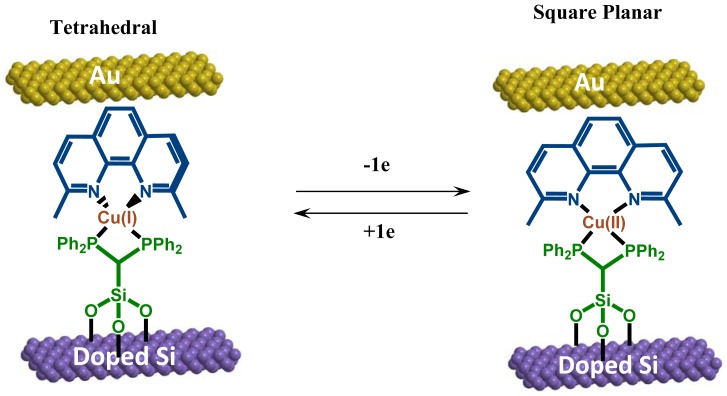
The schematic structure of the molecular rotor device composed of a Bisphenanthroline (bisP) stator, a copper metal axle, and a diimine rotator. The diimine ligand on top, 2,9-dimethyl-1,10-phenanthroline, rotates upon reduction and oxidation in the direction as illustrated with arrows.

One example of the surface mounted molecular motor is shown in Figure 4. Synthesized using a self-assembly approach, this molecular switch device is comprised of heteroleptic copper compounds covalently bonded to a highly doped silicon substrate. Each complex contains three subunits with a top contact electrode: a bifunctional stator (a bidentate ligand bonded to both a solid support (P^+^ Si) and a Cu axle), the metal axle (Cu), and a diimine rigid rotator (2,9-dimethyl-1,10-phenanthroline) on the doped Si (see Figure 4 for the top and bottom). Preparation of the heteroleptic copper system was carried out through covalent grafting of a stator monolayer onto the hydroxylated surface of a P^+^ Si substrate using silanol bonds [88]. The stators were then used to chelate a copper metal axle that subsequently bonds to the rotator diimine subunit. The device was completed by deposition of a Ti/gold film through a shadow mask on top of the molecular layer to form the top electrode. This copper compound exhibits two discrete, redox-dependent conformational states, Cu(I) and Cu(II). The Cu(I) form has tetrahedral geometry (on the left of Figure 4) while the Cu(II) form is square planar (right side of Figure 4) [57].

The compounds undergo a one electron redox-induced rotational conformational change depending on the oxidation state of the copper metal. Interconversion between these two states provides the basis for a controlled, bistable nanoswitch.

In order to confirm redox-dependent conformational switching, the authors took absorption spectra for both the bis(1,10-phenanthroline) Cu(I) and bis(1,10-phenanthroline) Cu(II) complexes in methylene chloride. An absorption peak, corresponding to a metal-to-ligand charge transfer (MLCT) indicative of tetrahedral geometry, was observed near 450 nm for the Cu(I) complex but not for the Cu(II) complex. Two additional peaks observed in both Cu(I) and Cu(II) complexes and centered at approximately 280 nm and 340 nm are attributed to ligand-to-ligand transitions, *i.e.*, π to π*, of the chelating ligands. These transitions and their corresponding energy states can be extracted from the peak positions to aid in constructing the proposed band diagram to be described later in Figure 8.

**Figure 5.**
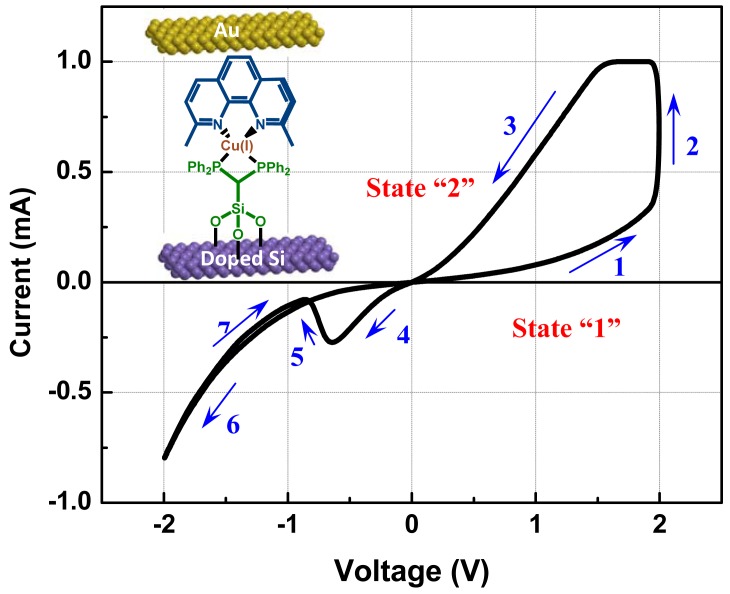
I-V characteristics of monolayer devices with the bisP stator. State “1” represents the low conductivity state, while state “2” is the high conductivity state. The turning voltage between state “1” and state “2” is determined by the redox energy of the copper system. The difference in value of the turning voltage between the positive and negative ranges is due to the different contact energy barriers of the two electrodes. The arrow shows the sequence of voltage scans. Arrows “1” and “3” correspond to the band diagrams in Figure 8, (**a**) and (**b**), respectively, while “4” and “7” correspond to (**c**) and (**d**).

**Figure 6.**
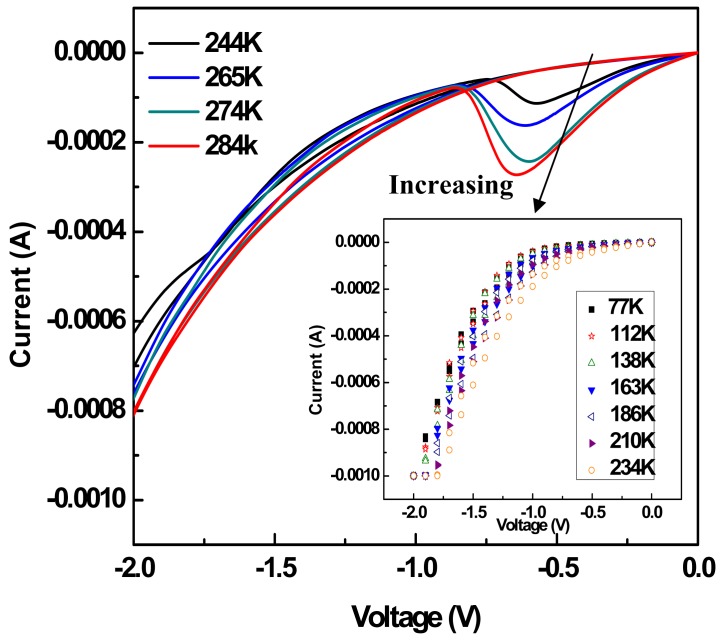
Temperature dependence of monolayer rotor devices with the bisP stator from 77 K to room temperature. In the range of the temperature higher than 244 K, the measured current in the NDR region increases along with temperature. In the range of temperature lower than 244 K, the NDR disappears and the measured currents do not show a significant dependence on temperature as shown in the inset.

I-V characteristics of the monolayer device are shown in Figure 5. In this case, the silicon substrate represents the system ground and positive bias corresponds to electron injection from the top gold electrode to the Cu(I) axle while negative bias corresponds to electron injection from the Si-OR contact (see also Figure 8(b)). The bias sweep direction followed the arrows numerically as shown in Figure 5. Unlike previously reported devices, the NDR observed here disappeared after reversal of the sweeping direction in the negative voltage regime at both macroscopic devices and microscopic CAFM (Conductive Atomic Force Microscopy) measurements (arrow 7) [89]. At room temperature (RT), the peak current density is approximately 0.1 A/cm^2^ with a peak-to-valley ratio (PVR) near 5. This RT-PVR exceeds that observed in typical heterostructure resonant tunneling diodes. The author also performed temperature dependent I-V measurements of the device from 77 K to room temperature as illustrated in Figure 6, which shows the NDR effect disappeared at below 244 K.

**Figure 7.**
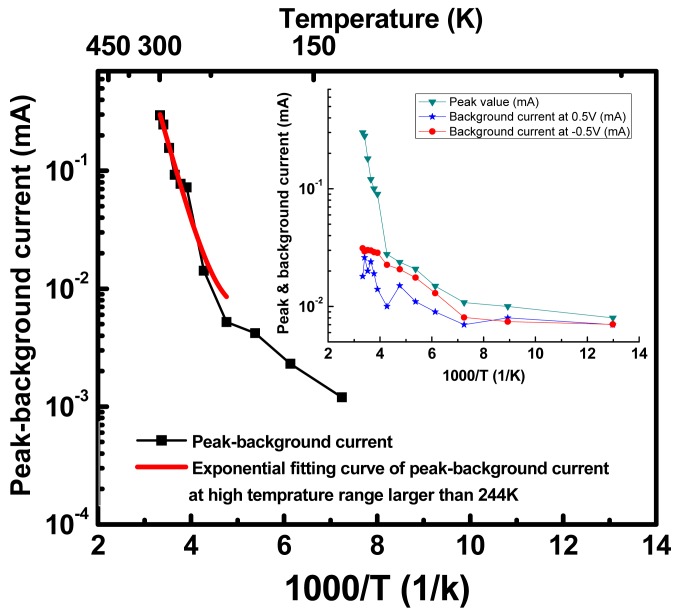
Arrhenius plot of the peak minus background current. The activation energy of rotation was extracted to be 0.3 eV by fitting curve of the measurement data higher than 244 K. The inset shows both the peak current and the background current at 0.5 V and −0.5 V. The temperature independence of the background current implies tunneling transport of electrons [90].

The results have been further analyzed to extract the activation energy. From the well-known Arrhenius equation below:

(1) $$E_{a}=-R{\left(\frac{\partial \ln\Delta I}{\partial (1/T)}\right)}_{P}$$

where E_a_ is the activation energy, Δ*I* is the peak minus background current, which is related to the rate constant of chemical reactions, T is the temperature and R is the gas constant. Curve fitting of the data for the temperature range of T > 244 K shown in Figure 7 yields a rotational activation energy of approximately 0.3 eV, and this value is consistent with the theoretical quenching energy of the rotation in solution. This solid agreement between the extracted activation energy and the theoretical quenching energy supports the proposal that the observed NDR effect in this device can be attributed to rotational motion within the molecular layer.

**Figure 8.**
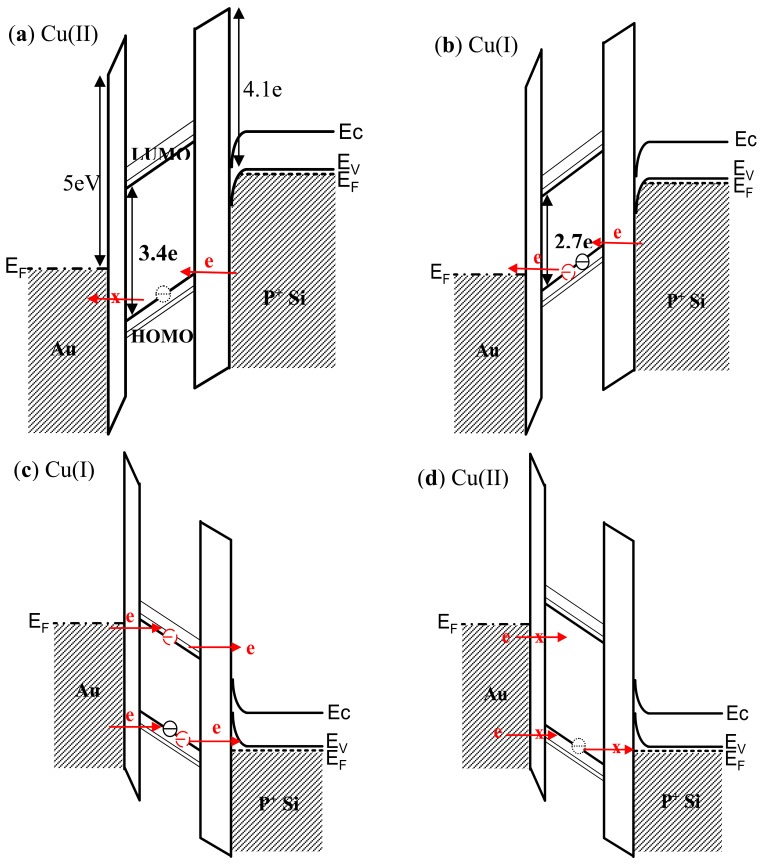
Band diagram of (**a**) low conductivity state of Cu(II) system under positive bias; (**b**) high conductivity state of Cu(I) system under positive bias; (**c**) high conductivity state of Cu(I) system under negative bias; and (**d**) low conductivity state of Cu(II) system under negative bias.

The proposed mechanism for the observed device I-V characteristics is based on an electron tunneling induced molecular rotation that serves to modify the band diagram of the active molecular layer. Absorption spectroscopy and DFT calculations have been employed in an attempt to reconcile the sequence of carrier transport processes and the role of energy states that have formulated the proposed band diagram presented in Figure 8. Application of a bias potential to the top gold electrode with respect to the grounded P^+^ Si substrate shifts the energy position of the molecular state relative to both gold electrode and P^+^ Si band-edges. As the rotary motion around the molecular axle is controlled by electron transfer, a portion of the applied potential is used to initiate the redox process within the molecular layer. First, calculations of the band gaps for the Cu(I) and Cu(II) complexes by TD-DFT yielded values of approximately 2.7 eV and 3.4 eV, respectively. Additional calculations of the molecular redox energy produced a value of 1.05 eV, which is of the same magnitude as the 0.7 eV difference of the band gaps between the Cu(I) and Cu(II) complexes. The 0.35 eV difference between these two values may be attributed to the rotational energy contribution. Finally, the electron affinity of the Cu(I) complex is estimated to be 3.7 eV, which anchors the LUMO energy state with respective to the gold Fermi level (see Figure 8).

The switching process begins with the low conductivity state of the Cu(II) complex under a positive bias as shown in Figure 8(a). The device remains in the low conductivity state until one electron from the P + Si substrate tunnels through the energy barrier and charges into the HOMO state of the active molecular layer. The resulting reduction of Cu(II) to Cu(I) shifts the relative position of the HOMO energy states upward and serves to shrink the band gap. As the conduction path for electrons is now open in the high conductivity Cu(I) state, current flow increases as seen in Figure 8(b) (the I-V data was shown in Figure (5). Upon application of a negative bias to the gold electrode, the conducting channel of the Cu(I) complex remains open and results in current flow through both HOMO and LUMO states as shown in Figure 8(c). Subsequent oxidation of Cu(I) to Cu(II) occurs when one electron tunnels out of the HOMO state and discharges the active molecular layer. This process alters the band structure and increases the band gap due to a reduction in Coulomb energy by the removal of one electron as seen in Figure 8(d). Current flow then reduces, resulting in NDR. The return trace does not show NDR behavior as the conduction path is closed. Continuing toward more negative bias produces an exponential increase in the measured current with respect to the applied electric field, which is therefore attributed to direct tunneling between the two electrodes. (*i.e.*, background leakage).

An additional attractive property of functional molecules based on rotational motion is their fast operational speed. In most cases, previous studies have focused on solution-based molecular rotors. With the perspective of exploiting the switching speed in mind, the rotation speed of the molecular rotor on P^+^ Si substrate was estimated. Since this molecular rotor is sufficiently large, the rotation speed can be estimated classically based on the kinetic energy of rotation and rotational inertia. As the angle between the Cu(I) and Cu(II) systems is 90 degrees, the rotation speed can be estimated as 1.4 ps, ignoring acceleration components of rotation during the beginning and the end of the movement. This value is reasonable as compared to previous simulation results of rotation in solution giving a speed of 0.5 ps [87].

To achieve logic functions, the molecular switches have to be able to be compatible with conventional CMOS manufactory technology. From this perspective, the surface mounted molecular device is the most likely candidate for the molecular electronics.

## 5. Scalability, Applications, Challenges and Outlook

As we have described above, this review deals with molecular rotors from the point of view of their potential utility for molecular switches and logic devices. In gases, liquids, and certain solids, entire molecules can rotate as a whole around independent axes freely, but they cannot be used as a stable electrical device and furthermore certain process flows have to be developed to be compatible with the traditional CMOS technology; so we restrict the scope of this section to the surface mounted molecular rotors, in which groundwork was laid down for the current developments [91–93]. As such, we will provide our analysis related to such rotation and focus our attention in the context of nanoscience which is relevant to the ultimate construction of molecular-size mechanical structures that might perform useful functions.

### 5.1. Scalability

In the advent of nanoelectronics, the question of inherent physical limits as well as possible technological barriers to scaling is among the most important. In this section, we will start with discussions on the fundamental physical limits of scaling; then we focus on the scalability of molecular rotor devices based on the redox assisted electron tunneling transport.

It was pointed out that the logic device of the size below 5 nm may preferably use a state variable that has a heavier effective mass than that of an electron [94]. In principle, the described surface mounted molecular rotor has an effective mass of approximate 4.5 × 10^−25^ kg, while that of free electron is 9.1 × 10^−31^ kg, four orders of magnitude higher than a free electron. Therefore the molecular rotor device can in principle be scaled down to below 5 nm. In fact, the designed monolayer molecular rotor device discussed in the last section is on the order of 3 nm scale. However the transport of this device is still controlled by electron tunneling instead of the atomic molecular tunneling if an electrical field is applied even though the tunneling barrier height may be larger than Si, for example. Nevertheless as to be discussed later, the redox energy adds additional tunneling barrier height.

As we discussed in the last section, the surface mounted molecular rotor is based on a redox-assisted electron tunneling transport model for the device operation. An applied electric field can induce the rotation associated with the redox reaction, thus change the energy barrier height and thus alters the availability of the states to be tunneled to. When the device operates at the Cu(II) state, the energy barrier is increased and electron transport only happens through a barrier to access the available states. Conversely when the device operates at the Cu(I) state, the energy barrier becomes lower and, the accessible states are readily available; thus electrons can easily tunnel through it. In general, the current at the Cu(II) case is lower and strongly unidirectional since the barrier behaves like a Schottky barrier due to the use of Si substrate; while the current at the Cu(I) case is larger and bidirectional, showing close to ohmic behavior.

The transition between the Schottky and tunneling modes is achieved by simply changing the polarity of the bias, which activates the redox reaction associated with the rotation movement. Thus the I-V data shows a nonlinear behavior with hysteresis as well as giving rise to negative differential resistance as described in Figure 5. These characteristics can be used to make two state switching devices, such as memory or logic elements for information processing applications.

It is important to note that the operation of this device is based on both electron transport and Redox reaction. Although the ultimate limiting factor of scalability of this device is still the leakage current as a result of the quantum tunneling of electron, the energy required by the redox reaction adds an additional energy barrier to the total tunneling barrier so that the probability of the tunneling becomes smaller. Due to the redox reaction, the scalability of this redox-assisted tunneling device should be better than the pure electron tunneling devices, in term of minimizing the leakage.

**Figure 9.**
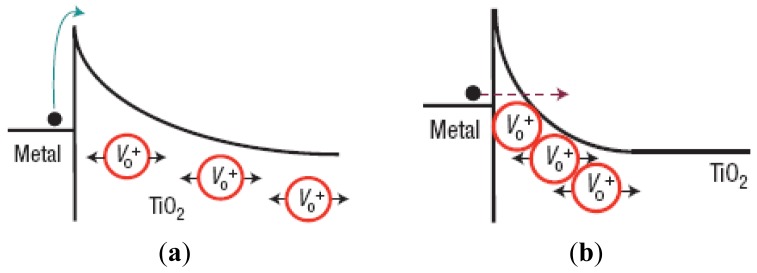
Energy barriers usually referred to a switch made by sandwiching a layer of titanium dioxide between two metal electrodes [98]. The energy barrier at the metal/oxide interface can be changed by using an electric field to move the positively charged oxygen vacancies (red) in the oxide layer away from the interface (**a**) or closer to it (**b**). For (**b**), the depletion width (tunneling width) is reduced to enhance electron current flow [99].

The suggestion that heavier particles might be preferred for nanoscale devices has attracted the attention of several research groups [94–96]. A number of memory concepts based on ion–migration effects in solids are currently being explored and studied for the scaling limits [94]. However, the improvement of the scaling limit of this type device is due to the energy barrier adjustment by ion migration as shown in Figure 9. The essential concept is, however, exactly similar to the redox-assisted tunneling device we described in the last section, in the sense that the barrier characteristics altered.

Further improvement of the scalability of this surface mounted molecular rotor can be achieved by replacing the metal electrode with a solid electrolyte. The idea is to build a pure ion transport system. It is important to carefully choose the solid electrolyte which determines the energy barrier as well as transport ions that are responsible for the current flow where a low bias is applied to move ions while electron tunneling does not occur. A mimic system may be human brain [97]. In human brain, the distribution of Ca ions in dendrites may represent a crucial variable for processing and storing information. Ca ions enter the dendrites through voltage gated channels in a membrane, and this leads to rapid local modulations of the calcium concentration within dendritic trees. Based on the brain analog, the binary state can be realized by a single ion that can move into one of two defined positions, separated by the barrier with voltage controlled conductance.

### 5.2. Issues and Challenges

Although specific organic molecules have their advantageous electronic properties, the reliability is always a challenge. The problem may be due to poor material uniformity because of inherent impurities, material interfaces, and electrode contacts. Therefore, the inherent characteristics of these molecules are easily masked by effects from the electrode materials and experimental conditions. Great cares must be exercised in attributing mechanisms and in constructing models to the observed switching behaviors and thus critical control experiments are required to obtain fundamental physical understanding. Once we fully understand the particular switching mechanism, studies of reliability, including a thorough investigation of all conceivable failure mechanisms, and subsequent development of optimization steps to correct them can follow.

The heat dissipation is another issue to be considered. The specific heat capacity and thermal conductivity of materials affect the device performance and limit the scalability of the molecular device. Proper selections of materials for their favorable heat diffusion rates and for controlled material interfaces on the atomic scale may circumvent the heat dissipation problem to the point to allow for scaling beyond the ultimate tunneling limit.

### 5.3. Applications and Outlook

To date, most of resistive memory devices are based on the change of resistance caused by an applied external electrical field or current [100]. The basic requirements are that a device must possess at least two stable states, that they can be switched by an external stimulus, and that the states can be clearly readout. Some most common devices using the resistive switching as their principal operational principle are, for example, resistance switching RAM, or PRAM. The switching mechanisms of these devices exploit a local metal-to-insulator transition rather than a conformational state change. Among various device structures showing electrically induced resistive switching effects, metal-insulator-metal (MIM) systems have been proposed as one of future attractive non-volatile memory technologies because of their scalability. In general, the insulator in the MIM chosen maybe from a wide range of binary, multinary oxides and chalcogenides as well as organic compounds, and the metals may be selected from a similarly large variety of metals, including conducting metals and conducting non-metals such as highly doped semiconductors; different metals are often used for the two sides of MIM.

A number of organic MIM devices have been reported to show resistive switching. Among the earliest work, Gregor [101,102] used the 10–25 nm glow-discharge-deposited polydivinylbenzene as the organic insulator. These devices showed several orders of the magnitude in the resistance change at 1–2 V. Switching in other organic films formed from small molecules has also been observed. Among the latter are anthracene [103], pentacene [104], 8-hydroxyquinoline aluminum (Alq_3_) [105,106], N,N′-bis(3-methylphenyl)-N,N′-diphenyl-1,1′-biphenyl-4,4′-diamine (TPD) [107], 2-amino-4,5-imidazoledicarbonitrile (AIDCN) [108], several fluroscent dyes with various electron withdrawing sub-stituents [109,110], and motor-molecules such as rotaxane [111,112]. Reports on these above devices already discussed some experimental difficulties in identifying intrinsic switching mechanisms; for example, asperities in the bottom electrodes, dust particles, pinholes in the organic layer, and shorting paths induced by deposition of the top electrode can all contribute to non-reproducible behaviors and lead to switching behaviors that are obscured from the intrinsic properties of ideal systems. Thus it is a challenging task to make a sensible comparative study and to assign a correct switching mechanism to each molecule system.

The redox-based nanoionic memory becomes attractive to the semiconductor industry because experimental demonstrations of scalability, retention and endurance are encouraging [113,114]. The operation of this type is based on a change in resistance of a MIM structure caused by ion (cation or anion) migration combined with redox [115,116]. Often, the conduction is of filamentary nature. Usually a highly conductive filament connecting the metal electrodes is formed at the ON state [117]. Upon reversal of polarity of the applied voltage, a dissolution process of these filaments takes place, resetting the system into the OFF state. If this effect can be controlled, the reliability can be established, and variability is assessed, memories based on this bi-stable switching process may be scaled to very small feature sizes. The switching speed is limited by the ion transport. Many details of the mechanism of the reported phenomena are still unknown. Developing an understanding of the physical mechanisms governing switching of the redox memory is a key challenge for this technology.

Although the devices we discussed above are all two terminal devices. In principle, a three terminal device could be designed with a barrier height controlled by a gate similar to conventional Field Effect Transistor (FET) devices. Such approach could reduce the leakage current due to the presence of an additional energy barrier as well as the use of heavier particles. Proper selections of materials for their electron affinity and controlled material interface on the atomic scale may also allow this device for going beyond the ultimate tunneling limit of electron and offers a potential to realize future non-volatile memory or logic devices for information processing applications.

To achieve these devices comparable to molecular devices of the size scale of <5 nm, the cost of conventional top down lithography may become prohibitively high for manufactory of these devices of integrated circuits. The patterning size variability due to the presence of subatomic particles and processing fluctuations at this scale increases the rate of faults. Beside to these fabrication challenges, nanoscale circuits also likely contain defects so numerous that some forms of defect tolerance will be necessary to achieve acceptable yields.

**Figure 10.**
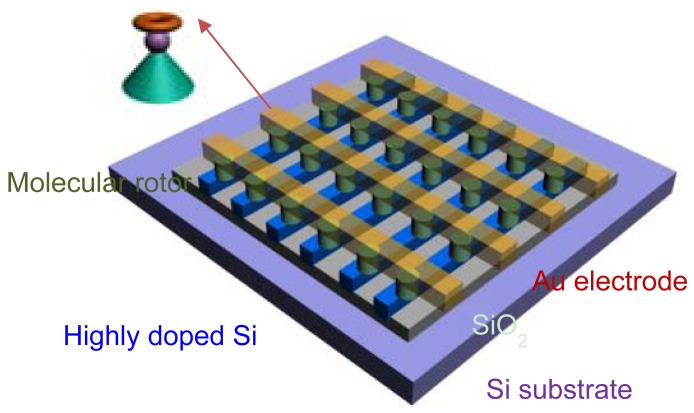
Schematic view of a nanoelectronic crossbar architecture. Junctions (molecular switch) may be independently configured to behave as electronic devices.

The above challenges of nanoscale devices in general and the yield issues associated with the molecular devices described above lead us to address on configurable crossbar architectures [118–123], where each cross point within two cross lines can be independently configured to activate an electronic device, such as resistive switch. As we mentioned in the last section, the two terminal molecular rotor device we studied can be easily implemented into this kind of cross bar architecture by overlaying two nanowire electrode arrays, so that a switch is formed at each crosspoint as shown in Figure 10.

The crossbar structure possesses many attractive features as it offers the highest possible device density and the simplest interconnect configuration that still allows for external access to each nanodevice [124]. It is also one of the easiest structures to build using other nanofabrication methods such as nanoimprinting lithography and self-assembly methods from the bottom up [125], saving today's industrial patterning technology [118,126,127]. Their high degrees of redundancy offer a simple strategy for defect tolerances. The regularity of these structures makes them easy to integrate devices and analyze fault tolerances.

Hybrid nanocrossbar/CMOS architectures have also been proposed to complement the limited functionalities of two-terminal switches by integrating with CMOS components to expand their functionalities [128–130]. It has been shown that hybrid crossbar/CMOS memories using such approach can offer a terabit potential density and a sub-100 ns access time. Furthermore, hybrid logic circuits based on the crossbar/CMOS structures can offer functional densities by at least two orders of magnitude higher than that of the conventional CMOS using the same design rules [130].

If a crossbar structure is referred to as a passive matrix architecture, the two-terminal molecular device, however, can also be integrated to become active matrix architecture by introducing a select transistor at each node by which the storage cells can be decoupled for those not addressed. This concept significantly reduces crosstalks, interferences and disturbing signals in the conventional crossbar system.

Overall, the concept of molecular memory emphasizes extreme scaling; in principle, one bit of information can be stored in the space of a single molecule. Because of their small size, very dense circuits could be built, and bottom-up self-assembly process could be applied to augment top-down lithography fabrication techniques. Computing with molecules as circuit building blocks is an exciting concept with several desirable advantages over conventional circuit elements. However, referring to the 2011 ITRS on emerging research devices, the molecular memory device still stands at a long term research state because of our limited understanding of the phenomena accompanying molecular switching, where currently many questions remain. In addition, the variability and reliability will need to be established.

In summary, there are a lot of interesting works in this field under progress such as ferroelectric molecular rotors [131], design of molecular rotors on metal surfaces [132,133], and others [134–136]. However, our paper is limited in its scope to describe the molecular rotors from the point of view of their potential utility for molecular switches and logic devices. Due to their advantages of scalability, highly integratable characteristics and the ability to self-assembling processes, these molecular machines hold a potential for memory and logic applications. The applications may begin with specific areas such as flexible and printed electronics. The challenges are to achieve high reliability and high yield manufacturability.
